# The impact of sprint interval training with or without weight loss on substrate oxidation in adults: A secondary analysis of the i‐FLEX study

**DOI:** 10.14814/phy2.15684

**Published:** 2023-05-05

**Authors:** Dawson Nancekievill, Benjamin H. Colpitts, Ken Seaman, Martine Girard, Martin Sénéchal

**Affiliations:** ^1^ Cardiometabolic Exercise & Lifestyle Laboratory University of New Brunswick Fredericton New Brunswick Canada; ^2^ Faculty of Kinesiology University of New Brunswick Fredericton New Brunswick Canada

**Keywords:** body composition, exercise, metabolic flexibility, obesity, substrate usage

## Abstract

Endurance exercise training and weight loss (WL) have been associated with changes in fat oxidation. However, there is limited evidence investigating the impact of sprint interval training (SIT)‐induced WL on fat oxidation in adults. To investigate the impact of SIT with or without WL on fat oxidation, 34 adults aged 19–60 years (males, *n* = 15) took part in 4‐week SIT. SIT consisted of 30‐s Wingates starting with two intervals and working up to four interspersed with 4 min of active recovery. Fat oxidation was estimated via indirect calorimetry using a metabolic cart during submaximal cycling. Following the intervention, participants were classified into a WL group (weight change >0 kg) or a non‐WL group (weight change ≤0 kg). No difference in resting fat oxidation (*p* = 0.642) and respiratory exchange ratio (RER) (*p* = 0.646) were observed between the groups. There was a significant interaction for the WL group with increased submaximal fat oxidation usage (*p* = 0.005) and decreased submaximal RER over the duration of the study (*p* = 0.017). When adjusted for baseline weight and sex, submaximal fat oxidation usage remained significant (*p* < 0.05), while RER did not (*p* = 0.081). The WL group had higher work volume, relative peak power, and mean power than the non‐WL group (*p* < 0.05). Short‐term SIT elicited significant improvements in submaximal RER and fat oxidation (FOx) in adults that lost weight, which may be explained by an increase in work volume throughout SIT training.

## Graphical abstract

Short‐term sprint interval training (SIT) elicited significant improvements in submaximal RER and fat oxidation in adults that lost weight, which may be explained by an increase in work volume throughout SIT training.

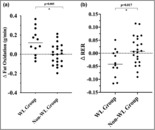

## 1 INTRODUCTION

More than one in three adults are living with obesity (Obesity Canada, 2021). This is of great concern as obesity has been associated with a reduced life expectancy of 5–10 years (Peeters et al., 2003) and reduced life free of disease by up to 13 years (Nyberg et al., 2018). This is not trivial since obesity and its chronic conditions have an estimated healthcare cost of approximately $9 billion per year (Obesity Canada, 2021). Although obesity is associated with chronic conditions, exercise and moderate weight loss (WL) appear to mitigate these effects (Brown et al., 2016; Feldman et al., 2017).

The onset of obesity‐related chronic conditions has been associated to the inability to efficiently alternate between substrate oxidation known as metabolic inflexibility (Battaglia et al., 2012). In a comprehensive review, Goodpaster and Sparks (2017) highlighted the need to address metabolic inflexibility as a means of treating obesity and its associated chronic conditions (Goodpaster & Sparks, 2017). Although studies have shown that impaired maximal fat oxidation (FOx) is associated with obesity, WL maintenance (maintained >10% WL) has improved maximal FOx (Dandanell et al., 2017). Specifically, Dandanell et al. (2017) observed 43% greater maximal FOx in a >10% WL group compared to 5%–10% WL group; however, this WL was achieved through a 12‐week lifestyle intervention (Dandanell et al., 2017). Similarly, diet‐induced WL has been shown to increase maximal FOx by 19% and significantly increase FOx per lean body mass by 29% in obese men (Tsujimoto et al., 2012). However, one randomized controlled trial found that WL alone was not a sufficient stimulus to increase fat utilization, but that exercise‐induced WL significantly increased submaximal FOx during moderate intensity exercise (Amati et al., 2008). Nevertheless, stimulating resting and submaximal FOx may potentially promote WL and improve health outcomes (Alkahtani et al., 2013; Astorino & Schubert, 2018; Dandanell et al., 2017; Gaitán et al., 2019; Goodpaster & Sparks, 2017).

Moderate‐intensity continuous training (40% to <60% of heart rate reserve) has been extensively studied as a means of achieving WL (Amati et al., 2008; Friedlander et al., 2007; Ho et al., 2012; Martin et al., 1993; Riebe et al., 2015; Skrypnik et al., 2015; Venables & Jeukendrup, 2008); however, time is often cited as a primary barrier to performing exercise (Jiménez‐Maldonado et al., 2020). Amati et al. (2008) showed that exercise‐induced WL was superior to diet or exercise alone for improving FOx (Amati et al., 2008). However, these results were observed in older adults (Amati et al., 2008) and the duration of exercise session was 45 min; thus not time‐efficient. It is imperative that time‐efficient forms of exercise, such as high‐intensity exercise, be investigated to determine its implication on WL and FOx, as lack of time is still consistently cited as a primary barrier to exercise (Cavallini, 2022). Although FOx has been shown to improve in as little as 2 weeks in individuals living with prediabetes or obesity when performing high‐intensity interval training (HIIT) rather than continuous training (Gaitán et al., 2019; Lanzi et al., 2015), a recent meta‐analysis demonstrated that the minimum length of exercise intervention to improve FOx is 4 weeks (Atakan et al., 2022).

In addition, a review suggested that both HIIT (exercise at 80%–95% of maximum heart rate) and sprint interval training (SIT) increase whole‐body FOx up to 26% while exercising and decreases respiratory exchange ratio (RER) by up to 20% (Astorino & Schubert, 2018). SIT is typically performed in bouts of 30‐seconds or less of maximal effort. Further evidence for the effectiveness of SIT at improving FOx was shown in two short‐term studies investigating the effects of SIT on young, active adults (Astorino et al., 2011; Burgomaster et al., 2008). However, the population of these studies limits the generalizability of these findings. The recent systematic review and meta‐analysis by Atakan et al. (2022) showed that although interval training interventions (HIIT and SIT) only improve FOx moderately more than moderate‐intensity continuous training, they typically require two times less time commitment. Together, with the previous evidence for maintained WL (Dandanell et al., 2017), exercise‐induced WL (Amati et al., 2008), and interval training (Astorino et al., 2011; Atakan et al., 2022; Burgomaster et al., 2008; Gaitán et al., 2019) for improving FOx, WL following SIT may serve to further improve FOx in a time‐efficient manner. Therefore, the primary objective of this secondary analysis of the i‐FLEX study was to further understand the role short‐term SIT‐induced WL has on resting and submaximal FOx and investigate their association with exercise performance, body composition, and metabolic outcomes. It was hypothesized that individuals that lost weight following SIT would significantly improve their submaximal and resting substrate oxidation rates.

## 2 METHODS

### 2.1 Study design and recruitment

**FIGURE 1:**
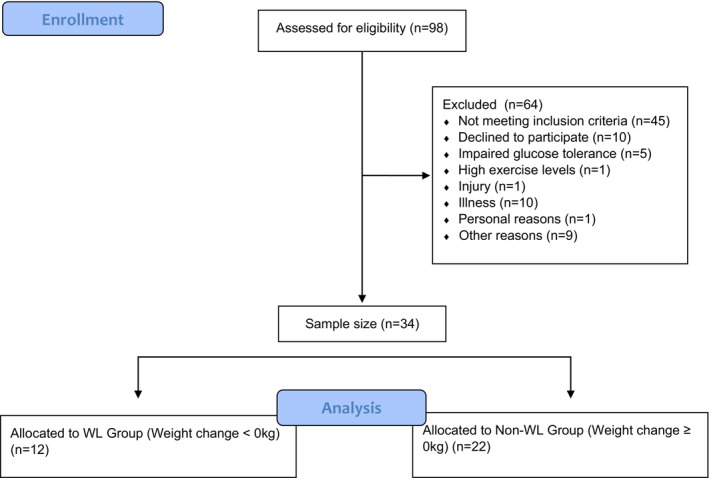
Participant flowchart.

This study was a secondary analysis of the “Effects of SIT on substrate oxidation in adults living with and without obesity: i‐FLEX study”, a clinical trial investigating changes in substrate oxidation and insulin sensitivity following 4 weeks of SIT between obese and non‐obese individuals (Clinical Trial #: NCT03527446) (Colpitts, Seaman, Eadie, et al., 2021). Thirty‐four adults aged 19–60 years (males, *n* = 15) took part in the study and were recruited through general community advertisement as well as by social media platforms (e.g., Facebook). An overview of participant recruitment can be seen in Figure 1. For 1 week, participants underwent baseline testing throughout three separate visits. Subsequently, participants commenced 4 weeks of SIT within 1 week of the final baseline visit. Upon completion of the intervention, participants performed follow‐up testing within 72 h of their final intervention session. All testing and intervention visits were performed at the Cardiometabolic Exercise and Lifestyle Laboratory at the University of New Brunswick. All participants provided written and informed consent before agreeing to participate.

### 2.2 Exclusion criteria

Participants were excluded if they (1) were aged outside of the prearranged thresholds, (2) were unable to perform SIT, (3) were living with diabetes or impaired glucose tolerance, (4) took medication known to impact insulin sensitivity and carbohydrate metabolism (i.e., corticosteroids or atypical antipsychotics), (5) enrolled in a weight‐loss program or exceeding a 10% WL within the previous 6 months, and (6) took medication known to cause weight gain or WL.

### 2.3 Exposure variable—SIT Intervention

Participants performed 4 weeks of SIT using a Monark 874E Weight cycle ergometer. Participants attended three supervised SIT sessions per week, with the number of intervals increasing as follows: two in week 1, three in week 2, and four in weeks 3 and 4. This progression of intervals was chosen as similar progressions have been used in previous SIT interventions (Burgomaster et al., 2008; Gibala et al., 2006). Participants began each session with a 4‐minute warm‐up consisting of light‐intensity cycling before the first sprint interval. The work: rest ratio consisted of a 30‐s Wingate followed by 4 min of active recovery at the minimum amount of wattage attainable on the cycle ergometer (59 W at 60 revolutions per minute). To remain consistent with previous Wingate protocols, the drop load was 7.5% of the participants' total body weight (Bar‐Or, 1987; Driss & Vandewalle, 2013). Heart rate was recorded immediately following each interval via the Zephyr™ BioHarness and OmniSense system (Medtronic). Total work performed; minimum, mean, and peak power output; and fatigue index were also recorded throughout each SIT session. Fatigue index was determined as the difference between the peak and minimum power divided by the peak power and multiplied by 100 to give a percentage. Before leaving the laboratory, participants performed a 5‐min cooldown and had seated heart rate and blood pressure measured to ensure proper recovery.

### 2.4 Primary outcome measure—substrate oxidation

#### 2.4.1 Resting substrate oxidation

RER, as well as FOx and carbohydrate (CHO) oxidation were estimated from a resting metabolic rate (RMR) test using a TrueMax 2400 Metabolic Measurement Cart (ParvoMedics) and a canopy system. Before arriving at the laboratory participants were asked to fast for 12 h and refrain from exercise for 48 h. The RMR test was conducted in a private room with the lights dimmed. Participants were requested to lie supine and motionless on a therapeutic bench during the RMR test. Over 30 min, average measurements were recorded every 30 s. The final 15 min of RER measurements were used to estimate resting substrate oxidation. FOx and CHO oxidation were estimated from the average VO_2_ and VCO_2_ measures from the last 15 minutes of the RMR testing using the following formulas (Frayn, 1983; Péronnet & Massicotte, 1991):

(1) $$\mathrm{FOx}\left(g/\min\right)=1.695\times {\mathrm{VO}}_{2}-1.701\times {\mathrm{VCO}}_{2}$$

(2) $$\mathrm{CHO}\;\text{oxidation}\;\left(g/\min\right)=4.585\times {\mathrm{VCO}}_{2}-3.226\times {\mathrm{VO}}_{2}$$

#### 2.4.2 Submaximal substrate oxidation

Similarly, RER, as well FOx and CHO oxidation were estimated from a metabolic cart (ParvoMedics) during an acute bout of exercise. Participants were required to follow a specific dietary protocol and refrain from any exercise for 48 h before the assessment. The standardized breakfast consisted of a set volume of Cheerios, milk, and orange juice consumed 2 h before the laboratory visit. The breakdown of macronutrients was as follows: 4.76 g of fat (14.2% of total calories), 55.48 g of carbohydrates (73.4% of total calories), and 9.37 g of protein (12.4% of total calories). For standardization purposes, participants were instructed to record their food consumption the day before baseline testing to ensure the same food was consumed before posttesting. The dietary protocol was completed to limit the impact diet at baseline and posttesting may have had on submaximal substrate oxidation values.

Participants were asked to cycle at 80–90 revolutions per minute for 4–6 min at 50% of VO_2_ and heart rate reserve. Based on previous literature and pilot data acquired in the laboratory, steady‐state was defined as a heart rate = ± five beats per minute and relative VO_2_ = ± 1.2 mL/kg/min using 30‐s averages over a minute, respectively (Myers et al., 1990). The Zephyr™ BioHarness and OmniSense system (Medtronic) were used to record heart rate measurements. RER values from the final minute of steady‐state exercise were averaged to estimate submaximal substrate oxidation. Oxidation rates were estimated using the same formula as previously stated with the VO_2_ and VCO_2_ values from the final minute of steady‐state exercise.

### 2.5 Exploratory measures

#### 2.5.1 Anthropometrics

Waist circumference and BMI were measured using the CSEP protocol (Canadian Society for Exercise Physiology, 2013). Hip circumference was also measured. Height and weight were measured to the nearest 0.5 cm and 0.1 kg, respectively. A calibrated column scale (SECA® model #213) was used to measure height and weight. Participants stood as straight as possible with their feet together, arms at their side, and without shoes. Following an inhalation, their height measurement was taken. Participants' weight was measured while wearing minimal clothing (shorts and t‐shirt). For the circumference measurements, participants stood with their feet shoulder‐width apart and their arms crossed. Upon a normal expiration, their waist circumference was measured at the upper lateral border of the iliac crest (Canadian Society for Exercise Physiology, 2013). Their hip measurement was taken around the greatest gluteal protuberance. An anthropometric tape was used for hip and waist circumference measurements, recorded to the nearest 0.5 cm per the CSEP protocol (Canadian Society for Exercise Physiology, 2013).

#### 2.5.2 Physical activity

Piezo Rx Pedometers were used to determine physical activity levels for 1 week, with a pre‐determined threshold of 100 steps per minute constituting moderate‐intensity physical activity (Marshall et al., 2013; Tudor‐Locke et al., 2011). Moderate‐to‐vigorous physical activity (MVPA) levels were quantified in bouts of 10 minutes or more.

#### 2.5.3 Body composition

Fat mass and fat‐free mass were estimated using the BOD POD version 1.69 (COSMED). During the BOD POD test, participants wore tight‐fitting clothing (compression shorts, spandex, sports bra, bathing suit, etc.) and a bathing cap while sitting still and breathing normally. The Siri formula [% Body Fat = (495/Body Density)–450] was used to estimate participants' fat mass and fat‐free mass, based on their body density value. It has been shown that the BOD POD is a valid and reliable tool to estimate body composition as error scores generally range from 1% to 2.7% for body fat (Vescovi et al., 2001). In our laboratory the coefficient of variation in 11 men (*n* = 6) and women (*n* = 5), is 3.5% for body fat percentage and 0.8% for fat‐free mass (Colpitts, Seaman, Bouchard, & Sénéchal, 2021).

#### 2.5.4 Glucose and lipid profile

The CardioChek PA Analyzer was used to analyze glucose and lipid profile and has previously shown strong reliability and validity (Bastianelli et al., 2017; CRMLN and NCEP, 2018; Gao et al., 2016). The research staff started by cleaning the participant's left ring finger with an alcohol swab. Two micropipettes (40 μL for lipids and 15 μL for glucose) were then filled and transferred to the respective testing strip for analysis (PTS Diagnostics). Measurements included glucose levels, high‐ and low‐density lipoprotein (HDL and LDL) cholesterol, total cholesterol, and triglyceride levels.

#### 2.5.5 Cardiorespiratory fitness

Fitness level was assessed by performing a graded cycle ergometer exercise test using the TrueMax 2400 Metabolic Measurement Cart (ParvoMedics). The protocol consisted of cycling at 80–90 revolutions per minute beginning with 5 min of cycling at 50 W; then, resistance was increased by 25 W every subsequent minute until exhaustion. To estimate cardiorespiratory fitness, VO_2peak_ values were averaged over the final 30 s in 5‐s increments. Heart rate, RER, and rate of perceived exertion were also recorded throughout the test. A VO_2_ plateau or an RER ≥1.10 and a rating of perceived exertion ≥17 on the Borg scale served as termination criteria (Edvardsen et al., 2013).

### 2.6 Group stratification

**FIGURE 2:**
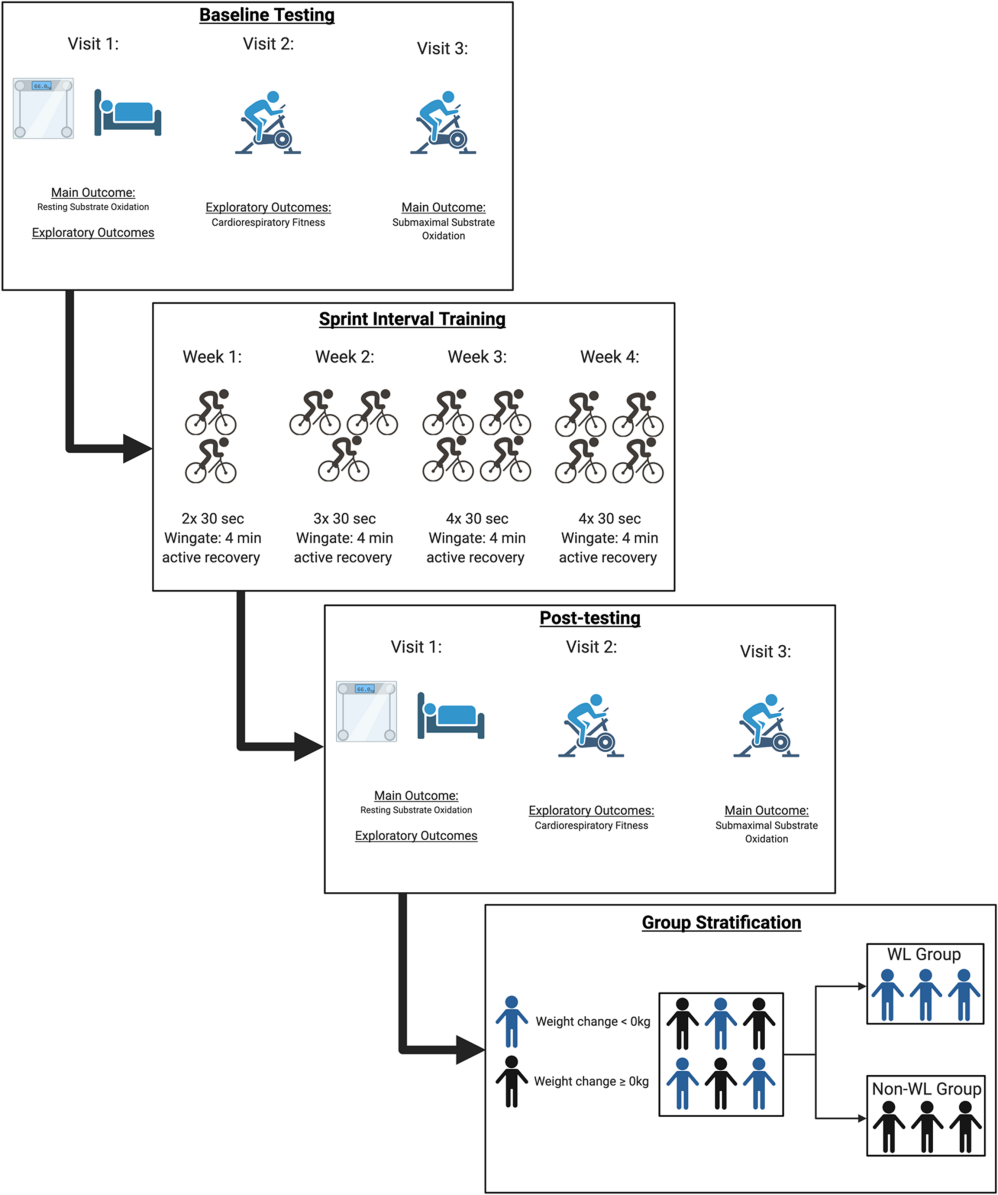
Study design and timeline.

Participants were stratified into two groups: the WL group or the non‐WL group. The WL group was defined as any reduction in body weight from baseline following the 4‐week intervention (weight change <0 kg), whereas the non‐WL group was characterized as either no loss or an increase in weight from baseline (weight change ≥0 kg) (Figure 2).

### 2.7 Statistical analysis

A power calculation for a repeated‐measures analysis of variance (ANOVA) was performed to determine the appropriate sample size for the main clinical trial, but no further analyses (Colpitts, Seaman, Eadie, et al., 2021). Accordingly, using G‐power software (Version 3.1.9.2, Germany) the sample size was calculated with a medium effect size of 0.4, an alpha of 0.05, and a power of 0.8. The total sample size per group was eight. Our final sample size was 17 per group (34 total), allowing us to account for potential dropouts and adjust for confounding variables (Linke et al., 2011).

Shapiro–Wilks tests were done to test for normality within the sample and a visual examination of the data confirmed normality. General characteristics are presented as mean ± SD for continuous variables and *N* (%) for categorical variables. Differences in baseline and posttesting values stratified by WL response were analyzed using paired sample *t*‐tests. Bivariate Pearson's correlations were performed to investigate whether changes in resting and submaximal FOx and RER were associated with performance and cardio‐metabolic outcomes. Subsequently, a repeated‐measures ANOVA was performed to determine whether there was a significant interaction effect between time and WL classification with resting and submaximal FOx and RER. The repeated‐measures ANOVA was adjusted for baseline weight and sex, which may impact WL following SIT as well as oxidation rates at baseline. SPSS version 16 and STATA 16.1 software (StataCorp) were used for data management and statistical analyses. A *p* ≤ 0.05 was considered significant.

## 3 RESULTS

### 3.1 Descriptive characteristics

Table 1 summarizes the descriptive characteristics of the sample stratified by WL group. There was no significant difference in age between the WL group; however, the proportion of females was significantly different for the WL group versus the non‐WL group: 16.7% versus 77.3%, respectfully (*p* = 0.001). A significant decrease in weight (97.5 ± 20.8 kg–96.2 ± 20.9 kg; *p* = 0.013), waist circumference (106.6 ± 17.2 cm–104.5 ± 16.9 cm; *p* = 0.007), and body fat percentage (33.4% ± 11.1–31.7% ± 10.8; *p* = 0.001) was observed in the WL group, which was significantly different compared to the non‐WL group (*p* < 0.05). Fat‐free mass levels were increased in both the WL (63.3 ± 8.7 kg–64.1 ± 8.2 kg; *p* = 0.049) and non‐WL (50.6 ± 10.2 kg–51.1 ± 10.4 kg; *p* = 0.030) groups. Systolic blood pressure was significantly changed in the WL (124.2 ± 15.4 mmHg–117.4 ± 12.3 mm Hg; *p* = 0.045) and non‐WL (110.9 ± 10.8 mm Hg–106.1 ± 12.2 mm Hg; *p* = 0.032) groups, while significant improvements in cardiorespiratory fitness were observed in the non‐WL group (*p* = 0.006), without significant differences between groups (*p* > 0.05).

**TABLE 1:** Characteristics of weight loss and non‐weight loss groups.

	Weight loss (*n* = 12)				Non‐weight loss (*n* = 22)			
	Pre	Post	Effect size (*g*)	*p*	Pre	Post	Effect size (*g*)	*p*
Female, *n* (%)	2 (16.7)	—	—	—	17 (77.3)^a^	—	—	—
Age (years)	44.5 ± 14.1	—	—	—	38.5 ± 11.3	—	—	—
Anthropometrics								
Weight (kg)	97.5 ± 20.8	96.2 ± 20.9	0.06	0.013	78.3 ± 22.1	79.2 ± 22.2	−0.04	0.000 ^a^
Body mass index (kg/m^2^)	30.5 ± 5.7	30.2 ± 5.7	0.05	0.051	26.9 ± 6.5	27.1 ± 6.5	−0.03	0.000 ^a^
Waist circumference (cm)	106.6 ± 17.2	104.5 ± 16.9	0.12	0.007	93.8 ± 16.0	94.1 ± 16.7	−0.02	0.470^a^
Body fat (%)	33.4 ± 11.1	31.7 ± 10.8	0.15	0.001	33.0 ± 12.9	32.9 ± 13.1	0.01	0.757^a^
Fat mass (kg)	33.8 ± 16.7	31.9 ± 16.3	1.31	0.001	27.5 ± 16.8	27.7 ± 17.1	−0.15	0.489
Fat‐free mass (kg)	63.3 ± 8.7	64.1 ± 8.2	−0.09	0.049	50.6 ± 10.2	51.1 ± 10.4	−0.05	0.030
Metabolic profile								
Resting SBP (mm Hg)	124.2 ± 15.4	117.4 ± 12.3	0.47	0.045	110.9 ± 10.8	106.1 ± 12.2	0.41	0.032
Resting DBP (mm Hg)	80.3 ± 10.3	77.7 ± 8.3	0.27	0.342	72.8 ± 11.6	71.7 ± 8.9	0.10	0.425
Total Chol (mmol/L)	5.2 ± 1.4	5.1 ± 0.8	0.08	0.573	5.1 ± 1.6	4.9 ± 1.1	0.14	0.171
HDL Chol (mmol/L)	1.3 ± 0.2	1.3 ± 0.3	0.00	0.836	1.5 ± 0.5	1.5 ± 0.4	0.00	0.721
Triglycerides (mmol/L)	1.5 ± 0.7	1.3 ± 0.5	0.32	0.126	1.4 ± 1.0	1.2 ± 0.7	0.23	0.106
LDL Chol (mmol/L)	3.2 ± 1.4	3.2 ± 0.9	0.00	0.804	3.0 ± 1.2	2.8 ± 0.8	0.19	0.166
Glucose (mmol/L)	5.0 ± 0.7	5.0 ± 0.7	0.00	0.870	5.0 ± 0.7	5.0 ± 0.7	0.00	0.762
Fitness level								
CRF (mL/kg/min)	32.5 ± 7.8	34.7 ± 8.6	−0.26	0.155	30.8 ± 9.6	32.4 ± 10.0	−0.16	0.006
Substrate oxidation								
Resting RER (VCO_2_/VO_2_)	0.80 ± 0.05	0.80 ± 0.04	0.00	0.871	0.80 ± 0.04	0.79 ± 0.04	0.25	0.575
Submaximal RER (VCO_2_/VO_2_)	0.96 ± 0.08	0.92 ± 0.06	0.55	0.030	0.93 ± 0.05	0.94 ± 0.05	−0.20	0.348^a^
Resting FO (g/min)	0.095 ± 0.024	0.096 ± 0.030	−0.04	0.929	0.083 ± 0.030	0.089 ± 0.031	−0.21	0.331
Submaximal FO (g/min)	0.117 ± 0.224	0.249 ± 0.165	−0.65	0.008	0.150 ± 0.120	0.142 ± 0.132	0.07	0.715^a^
Resting CHO (g/min)	0.138 ± 0.080	0.131 ± 0.051	0.10	0.770	0.102 ± 0.058	0.091 ± 0.046	0.19	0.446
Submaximal CHO (mg/min)	1.987 ± 0.654	1.756 ± 0.676	0.34	0.226	1.311 ± 0.532	1.347 ± 0.507	−0.07	0.470

*Note*: Variables are presented as means ± standard deviation.

Abbreviations: CHO, carbohydrate oxidation; Chol, cholesterol; CRF, cardiorespiratory fitness; DBP, diastolic blood pressure; FO, fat oxidation; HDL, high‐density lipoprotein; LDL, low‐density lipoprotein; RER, respiratory exchange ratio; SBP, systolic blood pressure.

^a^ Significant difference between groups. Alpha level at 0.05.

### 3.2 Changes in fat oxidation stratified by WL group

**FIGURE 3:**
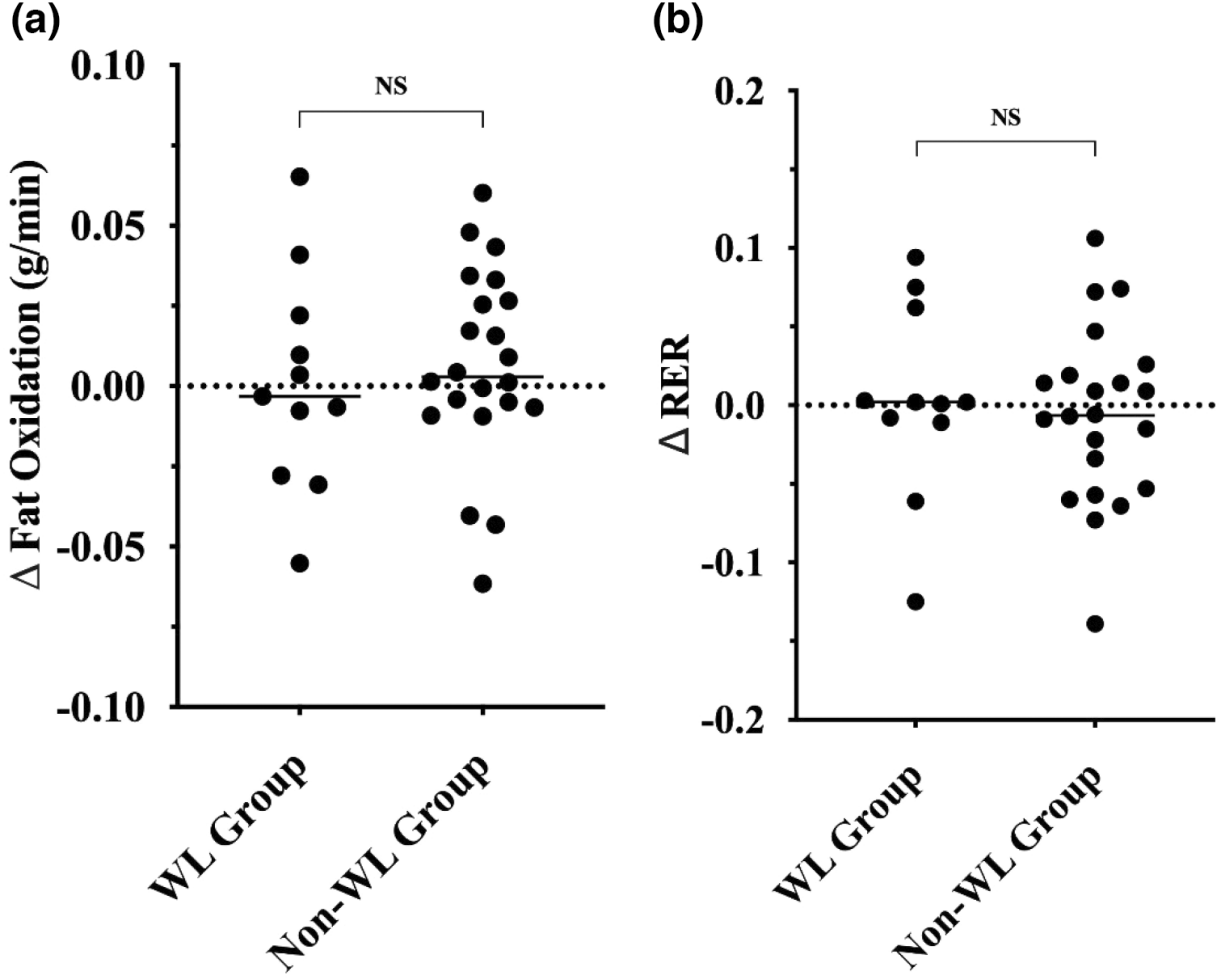
(a) Absolute change in resting fat oxidation. (b) Absolute change in resting respiratory exchange ratio. Data are presented as individual data points. Significant difference was considered *p* ≤ 0.05.

**FIGURE 4:**
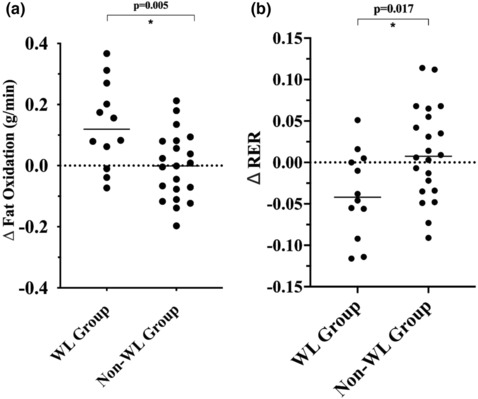
(a) Absolute change in submaximal fat oxidation. (b) Absolute change in submaximal respiratory exchange ratio. Data are presented as individual data points. Significant difference was considered *p* ≤ 0.05.

Baseline resting (RER: *p* = 0.997 vs. FOx *p* = 0.259) and submaximal (RER: *p* = 0.210 and FOx: *p* = 0.637) substrate oxidations were not statistically different between the non‐WL and WL group. Figure 3a,b present the changes in resting substrate oxidation (FOx and RER) following SIT stratified by WL groups. No differences were observed between WL groups for changes in resting oxidation measures (all *p* > 0.05). Figure 4a presents the changes in submaximal FOx while Figure 4b presents the changes in submaximal RER, following SIT stratified by WL groups. No improvement was observed for FOx or RER for the non‐WL group (all *p* > 0.05). The WL group displayed a significant increase in submaximal FOx (0.117 ± 0.224 g/min to 0.249 ± 0.165 g/min; *p* = 0.008) following SIT, which was significantly different from the non‐WL group (0.150 ± 0.120 g/min vs 0.142 ± 0.131 g/min; *p* = 0.005). Similar findings were observed for submaximal RER as the WL group experienced a significant improvement in RER (0.96 ± 0.08–0.92 ± 0.06; *p* = 0.030), which was also significantly different from the non‐WL group (0.92 ± 0.04 vs 0.94 ± 0.05; *p* = 0.017).

### 3.3 Differences in performance outcomes between weight response groups

Table 2 presents the differences between groups in performance outcomes throughout SIT intervention. The WL group had significantly higher work volume (65,267.2 ± 12,602.6 kgm vs. 47,061.7 ± 13,190.1 kgm; *p* < 0.001), higher relative peak power (11.5 ± 1.4 W/ffm vs. 10.4 ± 1.5 W/ffm; *p* = 0.039), and higher relative mean power (8.7 ± 1.0 W/ffm vs. 7.8 ± 1.0 W/ffm; *p* = 0.018) over the course of the 4‐week intervention compared to the non‐WL group. No group differences were observed for any of the other performance variables.

**TABLE 2:** Differences in performance variables between groups.

	Weight loss	Non‐weight loss	Effect size (*g*)	*p*‐value
Work volume (kgm)	65,267.2 ± 12,602.6	47,061.7 ± 13,190.1	1.37	<0.001
Peak power (W/ffm)	11.5 ± 1.4	10.4 ± 1.5	0.73	0.039
Peak power (W/bw)	7.6 ± 1.3	6.7 ± 2.2	0.45	0.179
Mean power (W/ffm)	8.7 ± 1.0	7.8 ± 1.0	0.88	0.018
Mean power (W/bw)	5.8 ± 1.2	5.0 ± 1.7	0.51	0.154
Minimum power (W/ffm)	5.5 ± 0.9	5.1 ± 0.9	0.43	0.348
Minimum power (W/bw)	3.7 ± 1.1	3.3 ± 1.2	0.33	0.369
Fatigue index (%)	52.7 ± 7.2	50.3 ± 6.1	0.36	0.298
Average heart rate (bpm)	161.4 ± 11.2	169.0 ± 10.0	−0.71	0.057
Percentage of maximum heart rate	92.0 ± 5.1	93.9 ± 5.7	−0.34	0.348

*Note*: Variables are presented as means ± standard deviation. Alpha level at 0.05.

Abbreviations: bpm, beats per minute; bw, body weight; ffm, fat‐free mass; W, watts.

### 3.4 Correlation between exploratory measures and Main outcomes

**FIGURE 5:**
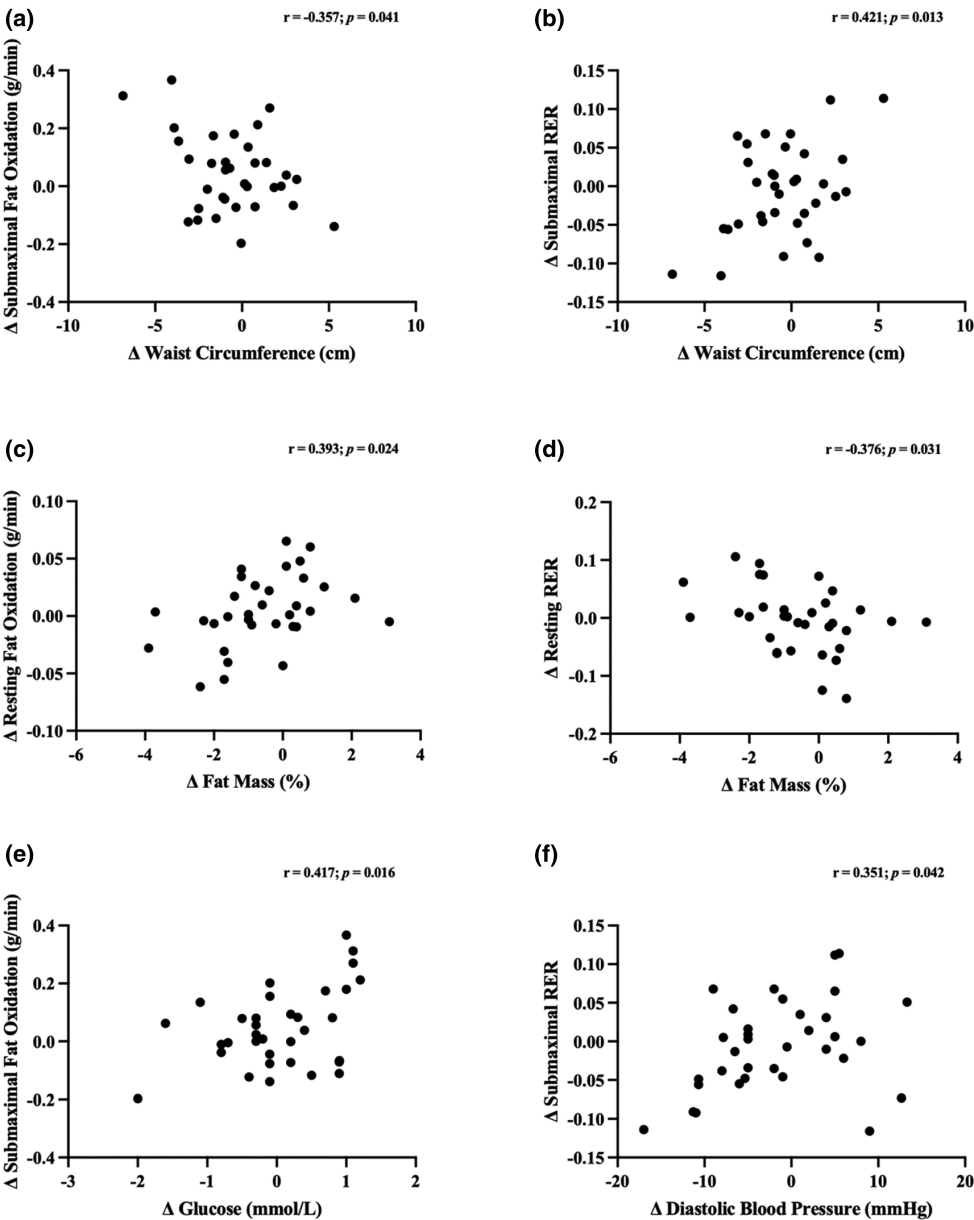
Correlation between main outcomes and exploratory outcomes for the whole sample: (a) Change in submaximal fat oxidation and waist circumference; (b) change in submaximal respiratory exchange ratio and waist circumference; (c) change in resting fat oxidation and fat mass; (d) change in resting respiratory exchange ratio and fat mass; (e) change in submaximal respiratory exchange ratio and diastolic blood pressure; (f) change in submaximal fat oxidation and fasting glucose.

Figure 5a–f presents correlations between changes in exploratory measures and substrate oxidation measures for the whole sample. Change in waist circumference was negatively correlated with change in submaximal FOx (Figure 5a: *r* = −0.357; *p* = 0.041) and positively correlated with changes in submaximal RER (Figure 5b: *r* = 0.421; *p* = 0.013) following SIT. Furthermore, change in percent fat mass was positively correlated with change in resting FOx (Figure 5c: *r* = 0.393; *p* = 0.024) and negatively correlated with resting RER (Figure 5d: *r* = −0.376; *p* = 0.031). Finally, change in diastolic blood pressure was positively correlated with change in submaximal RER (Figure 5f: *r* = 0.351; *p* = 0.042) while change in fasting glucose was positively correlated with change in submaximal FOx (Figure 5e: *r* = 0.417; *p* = 0.016).

**FIGURE 6:**
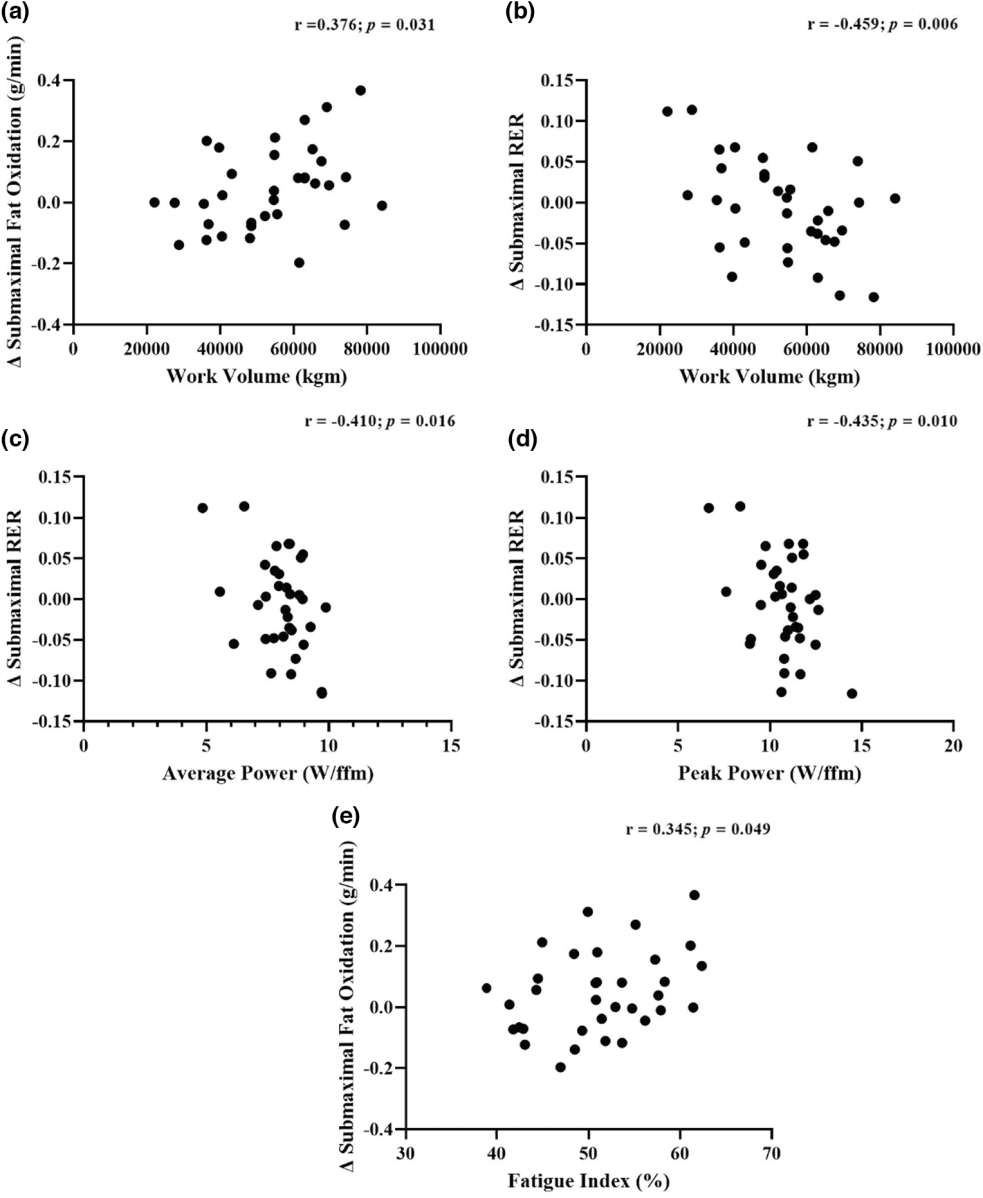
Correlation between main outcomes and performance outcomes for the whole sample: (a) Change in submaximal fat oxidation and total work volume; (b) change in submaximal respiratory exchange ratio and total work volume; (c) change in submaximal respiratory exchange ratio and average relative mean power; (d) change in submaximal respiratory exchange ratio and average relative peak power; (e) change in submaximal fat oxidation and average fatigue index.

Figure 6a–e presents correlations between changes in performance measures and substrate oxidation measures for the whole sample. Total work volume performed throughout SIT was positively correlated with changes in submaximal FOx (Figure 6a: *r* = 0.376, *p* = 0.031) and negatively correlated with changes in submaximal RER (Figure 6b: *r* = −0.459, *p* = 0.006). Additionally, change in submaximal RER was negatively correlated with both average power (Figure 6c: *r* = −0.410, *p* = 0.016) and peak power (Figure 6d: *r* = −0.435, *p* = 0.010) relative to fat‐free mass. Finally, change in submaximal FOx was positively correlated with fatigue index (Figure 6e: *r* = 0.345 *p* = 0.049).

### 3.5 Repeated measures ANOVA

No significant main or interaction effects were observed for either resting variable (all *p*s >0.05), suggesting that no difference in substrate oxidation changes were observed at rest between groups. A significant interaction effect was observed for submaximal RER [F(1,31) = 6.336, *p* = 0.017] and FOx [F(1,31) = 9.326, *p* = 0.005]. The interaction effect remained significant for submaximal FOx when adjusting for sex [F(1,31) = 6.007, *p* = 0.020], baseline weight [F(1,31) = 5.725, *p* = 0.023], and total work [F(1,31) = 4.234; *p* = 0.048]. However, no interaction effect was observed for RER following these adjustments (all *p*s >0.05).

## 4 DISCUSSION

The main objectives of this study were (1) to investigate the role of SIT with or without WL on resting and submaximal FOx, and (2) to investigate if changes in substrate oxidation were associated with exercise performance, changes in body composition, and metabolic profile following a 4‐week SIT intervention. The main findings of this study indicate that 4 weeks of SIT‐induced WL decreases RER and increases FOx during submaximal exercise compared to SIT without WL. Changes in FOx following SIT is correlated with exercise performance, changes in waist circumference, and fat mass. These findings support that when WL occurs following short‐term SIT, it is a viable time‐efficient method of improving substrate oxidation during exercise.

In line with previous research investigating changes in substrate oxidation following SIT or HIIT interventions (Alkahtani et al., 2013; Astorino et al., 2011; Atakan et al., 2021; Gaitán et al., 2019; Lazzer et al., 2017), we estimated substrate oxidation during submaximal exercise. The relevance of using submaximal exercise to quantify substrate oxidation is threefold. Because submaximal exercise has been used previously, it allows for our findings to be compared with previous research. Second, individuals spend most of their time during free‐living conditions at submaximal intensities shifting between carbohydrate and fat oxidation daily. Because of this, our results allow us to apply the influence of SIT‐induced WL on day‐to‐day conditions outside of exercise. Finally, quantifying substrate oxidation at maximal intensities using a metabolic cart has not been validated, limiting us to the use of steady‐state exercise.

Our data showed an increase in submaximal FOx following 4 weeks of SIT even after adjusting for confounding variables (sex, baseline weight, total work performed). This increase in FOx is in line with previous research examining high‐intensity exercise interventions and FOx (Astorino & Schubert, 2018). Gaitán et al. (2019) observed increased FOx at submaximal intensities following 2 weeks of HIIT (Gaitán et al., 2019). However, this increase in submaximal FOx was observed in a population of older adults living with obesity and prediabetes and the training intervention consisted of 3‐minute intervals at 90% of peak heart rate (Gaitán et al., 2019). Similarly, a 2‐week HIIT intervention consisting of 60‐s intervals significantly increased rates of FOx, decreased rates of CHO oxidation, and decreased RER in male adults living with obesity (Lanzi et al., 2015). Although these were short‐term interventions, time spent exercising is still greater than during SIT interventions. For example, RER was significantly reduced in age‐matched active men and women following only six sessions of SIT, suggesting improved FOx (Astorino et al., 2011). However, all participants from this study were young active adults and no WL was accounted for in their analysis. Similarly, a study of healthy young men and women observed an increased FOx and decreased carbohydrate oxidation during submaximal exercise following 6 weeks of SIT, similar to the traditional endurance training group (Burgomaster et al., 2008). Our results strengthen the body of literature suggesting that only 4 weeks of SIT is an effective method of improving FOx during submaximal exercise. Furthermore, our results, in combination with the results of Burgomaster et al. (2008), show that SIT is a time‐effective method of improving substrate oxidation. Their SIT group performed approximately 10 minutes of total exercise per week, whereas their endurance training group performed 4.5 h of exercise per week (Burgomaster et al., 2008). This discrepancy in time spent exercising highlights the efficiency of SIT versus endurance exercise for improving submaximal FO. Altogether, the present study builds on previous literature suggesting that training of short duration, including SIT, leads to improvements in submaximal FOx.

No significant improvements in substrate oxidation at rest were observed in either group. This result aligns with prior research that observed no significant change in RER following 4 weeks of SIT (Schubert et al., 2017). Our data strengthen these previous data by accounting for sex and WL, two factors believed to influence FOx (Berggren et al., 2008; Frandsen et al., 2021). Although the non‐WL group contained significantly more females than the WL group a recent review suggested that sex was not a meaningful moderator of the effects of SIT on FOx (Atakan et al., 2022). Likewise, it has been shown that menstrual phase does not impact peak FOx or maximal FOx during a graded exercise test in eumenorrheic women (Oosthuyse & Bosch, 2010) and that menstrual phase‐associated changes in exercise metabolism are inconclusive (Frandsen et al., 2020). Furthermore, oral contraceptive use has not been shown to affect free fatty acid concentration or substrate oxidation during exercise (Casazza et al., 2004; Isacco et al., 2012). To the best of our knowledge, most studies have investigated several modalities of exercise on resting substrate oxidation, but have not focused on the impact of SIT on resting substrate oxidation. For example, a randomized trial investigating the acute effects of exercise modality on resting energy expenditure and RER showed that HIIT lowered RER compared to other forms of exercise 60 min post‐exercise (Wingfield et al., 2015). The present study varies from prior research as most research has focused on HIIT or other modalities over SIT when investigating substrate oxidation at rest.

In our data, we observed that the SIT‐induced WL group demonstrated significantly greater performance variables throughout the 4‐week intervention. The WL group performed more overall work than the non‐WL group, which could have contributed to the improved submaximal FOx. In fact, previous studies suggest that FOx has been associated with training status and previous exercise status (Purdom et al., 2018). Therefore, it is possible that our SIT‐induced WL were previously trained; however, this is unlikely since the baseline cardiorespiratory fitness values were not different between groups. In addition, as individuals undergo adaptation via exercise they are able to oxidize fats at greater relative intensities which may have occurred during the present study, aligning our findings with previous literature (Lima‐Silva et al., 2010; Purdom et al., 2018). Similarly, negative correlations were observed between change in submaximal RER and average power and peak power suggesting that the higher the average and peak power over the course of the intervention the greater the level of FO. This could be due to the potential impact of exercise intensity on fat oxidation (Lima‐Silva et al., 2010). Participants in the present study that worked at a greater intensity during the Wingates due to greater drop‐weight relative to fat‐free mass may have improved substrate oxidation more than those that worked at a lower relative load. Altogether, our data support that SIT‐induced WL leading to enhance FOx is associated with greater performance metrics.

Significant correlations were observed for submaximal substrate oxidation and change in waist circumference; such that improvements in submaximal FOx was associated with a reduction in waist circumference, and a reduced submaximal RER was associated with a reduced waist circumference. These are in line with observations from a randomized, double‐blind, controlled nutrition‐based WL intervention where improvements in FOx and reductions in waist circumference was observed (Lightowler et al., 2019). Significant correlations were also observed for resting substrate oxidation values and change in fat mass percentage. As the change in resting FOx and RER increased, they were both correlated with an increased change in fat mass percentage. Similar relationships were observed by Doucet et al. (2000) between resting FO, RER, and fat mass; however, this was only observed in the women in their sample, whereas the WL group in the present study was primarily males (Doucet et al., 2000). Our data confirm and add to the literature as we show an association between resting RER and FOx and changes in fat mass percentage in both men and women.

Despite WL following SIT, the WL group increased their fat‐free mass levels. This data suggests that SIT protects against the loss of fat‐free mass typically associated with WL. This is of great interest since usually resistance training is recommended to preserve fat‐free mass (Campbell et al., 2009; Hunter et al., 2008). For example, in the intervention by Gaitán et al. (2019), a significant loss of fat‐free mass was observed in both the continuous and interval exercise groups (Gaitán et al., 2019). Similarly, following a 3‐week intervention with groups adhering to varying exercise intensities, fat‐free mass was significantly reduced following each of the interventions (Lazzer et al., 2017). Our findings are contradictory to previous findings showing that various forms of interval training induce losses in fat‐free mass; however, SIT can vary greatly from the various forms of HIIT, and SIT may be a sufficient stimulus to induce muscle hypertrophy.

Although the present study provides important insight on substrate oxidation and SIT‐induced WL, some limitations require acknowledgment. First, the study has a small sample size, which may have impacted the level of significance observed in the main statistical analysis. Second, participants may have only reached a VO_2peak_ rather than a true VO_2max_ measurement during the cardiorespiratory test, which may have impacted the relative exercise intensity for the submaximal substrate oxidation measurement. Third, in the main trial, MVPA was used as an exclusion criterion for the group living with obesity. This may have impacted the findings considering the original groupings were stratified differently than the current analysis. However, no differences were observed in baseline cardiorespiratory fitness levels and changes in fitness following SIT, which we believe is more important compared to MVPA levels. Previous training experience was not recorded for either group, which may impact the findings observed. Finally, baseline and postintervention testings were not necessarily matched for time of day which may serve as a limitation as there is evidence showing circadian rhythms may impact substrate oxidation rates (Sato et al., 2019). Nevertheless, the study is strengthened by its design allowing for the comparison of individuals that experience WL versus those that did not following SIT, which has not been performed prior to the current trial. Another strength of the current study is the identical dietary protocol at baseline and posttesting to mitigate any differences induced by diet prior to testing. In addition, SIT was supervised in a one‐on‐one environment by the same researchers over the 4‐week period, allowing the study to have a tightly controlled environment for the exposure variable. Finally, analyses were adjusted for confounding variables.

In summary, SIT‐induced WL resulted in decreased RER and increased FOx during submaximal exercise, while preserving fat‐free mass. Thus, it appears that SIT is a viable, time‐efficient manner to improve submaximal FO. This increase in FOx during submaximal exercise was correlated with improvements in body composition and increased work volume, peak power, and mean power. Future studies should focus on providing a better understanding of the underlying mechanisms by which SIT‐induced WL impacts oxidation rates.

## AUTHOR CONTRIBUTIONS

DN contributed to data analysis and drafting and editing the manuscript. BHC contributed to conceiving the main idea and design of the study, data collection and analysis, and drafting and editing the manuscript. KS contributed to conceiving the main idea and design of the study, data analysis, and drafting and editing the manuscript. MG contributed to drafting and editing the manuscript. MS contributed to conceiving the main idea and design of the study, data analysis, and drafting and editing the manuscript.

## FUNDING INFORMATION

This research project was supported from a research grant from the University of New Brunswick‐University Research Fund. Dr. Sénéchal was supported by an Establishment Grant from the New Brunswick Health Research Foundation (NBHRF), the University Research Fund, and Diabetes Action Canada. Benjamin H. Colpitts' salary was supported by funding from the Maritime SPOR SUPPORT Unit (MSSU) NBHRF as well as the Canadian Institutes of Health Research (CIHR). Dawson Nancekievill was supported by funding from the New Brunswick Innovation Foundation (NBIF) as well as the Canadian Institutes of Health Research (CIHR).

## CONFLICT OF INTEREST STATEMENT

The authors declare no conflict of interest.

## ETHICS STATEMENT

The project was approved by the University of New Brunswick Research Ethics Board (REB 2018‐058).

## Data Availability

Data generated or analyzed during this study are available from the corresponding author upon reasonable request.
